# Follicular Mucinosis: A Case Report

**DOI:** 10.7759/cureus.4746

**Published:** 2019-05-24

**Authors:** Adeyinka O Akinsanya, Jaime A Tschen

**Affiliations:** 1 Pathology, St. Joseph Dermatopathology, Houston, USA; 2 Dermatology, St. Joseph Dermatopathology, Houston, USA

**Keywords:** follicular mucinosis, alopecia mucinosa, mycosis fungoides, mucin

## Abstract

Follicular mucinosis (FM) is a rare disorder of the skin characterized by follicular degeneration due to the accumulation of mucin within the pilosebaceous unit, with associated inflammatory changes. We report a case of an 11-year-old female with widespread lesions showing distinct clinical and histological features of FM with a brief review of the literature.

## Introduction

Follicular mucinosis is a rare cutaneous mucinosis characterized by accumulation of mucin at the external root sheath and sebaceous glands [1]. It manifests clinically as follicular papules distributed on the trunk, proximal limbs, scalp, and face; it may also exist as indurated plaques [2]. FM has been described broadly as a primary benign condition, running an acute and/or chronic course, or as a secondary disorder to various other benign and malignant conditions. Skin biopsy and pathological interpretation are used to correctly identify and characterize the disease, and to differentiate it from other conditions.

## Case presentation

**Figure 1:**
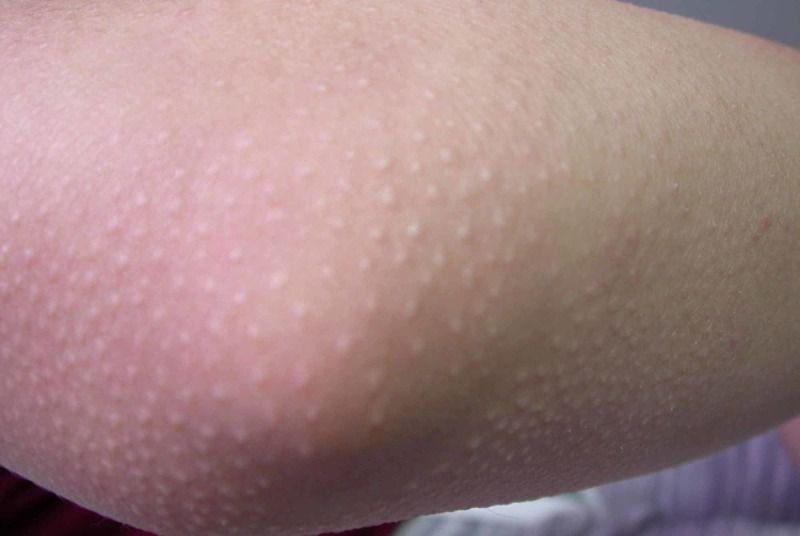
Clinical photograph of the elbow showing follicular papules.

**Figure 2:**
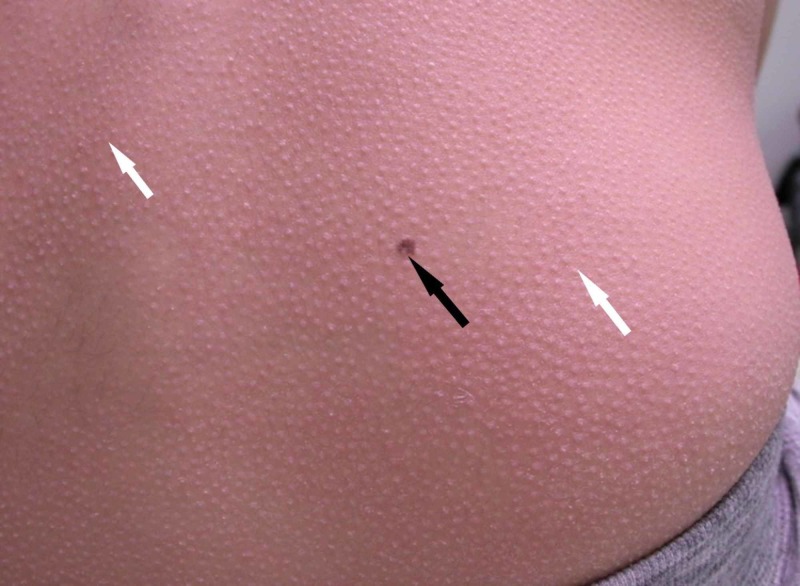
Clinical photograph of the back showing widespread follicular papules (white arrows) and an incidental melanocytic nevus (black arrow).

**Figure 3:**
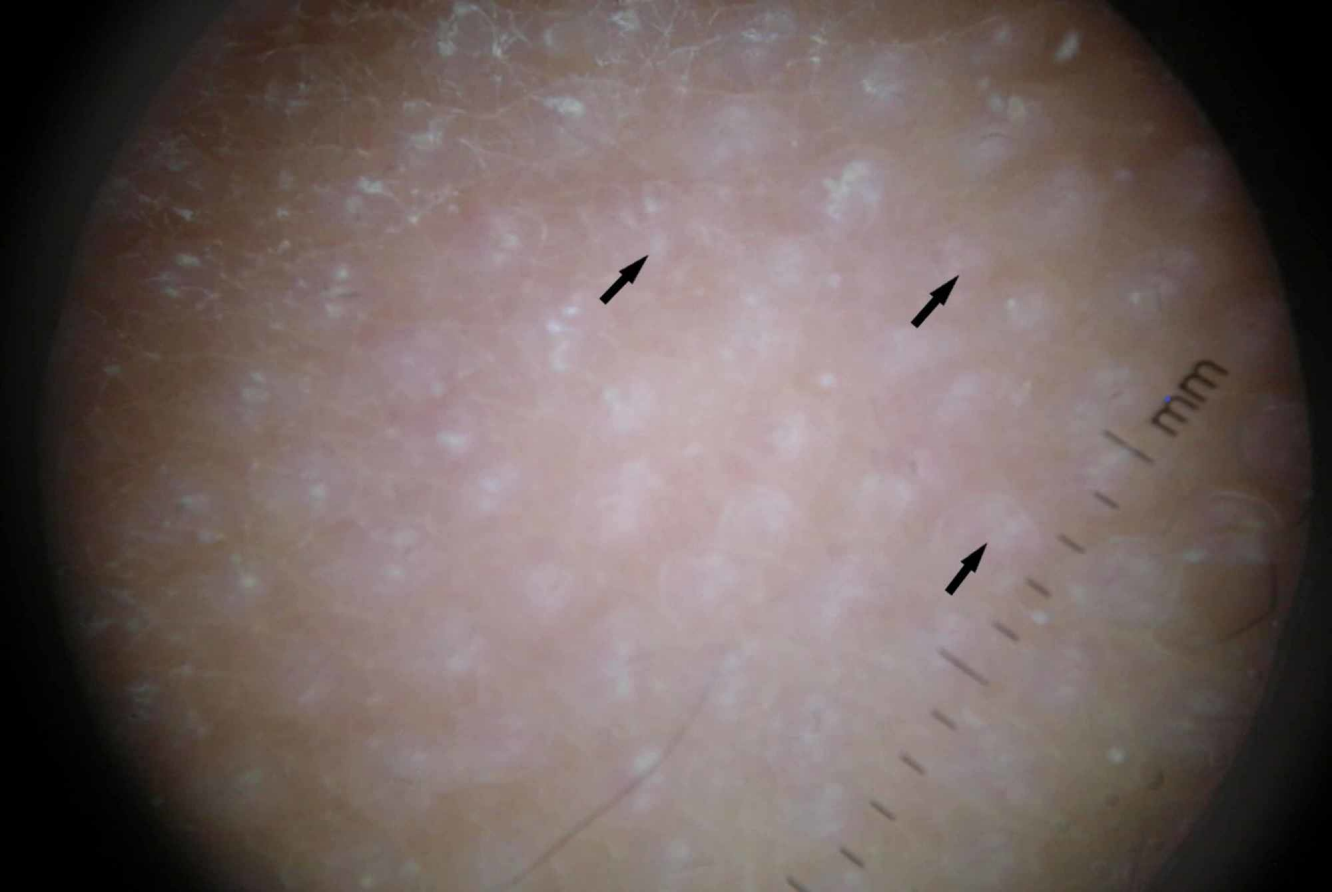
Dermatoscope view showing multiple papular lesions (arrows) on an erythematous base.

An 11-year-old female was referred to our center with multiple skin papules of 11 months duration. She was on no medications at the time. Past history was only significant for early puberty treated with gonadotropin releasing hormone agonists. Examination revealed widespread follicular papules which were located mainly on the elbows, knees, buttocks, and lower back (Figures 1-2). Close examination (including evaluation with a dermatoscope) revealed multiple small papular lesions on an erythematous base measuring approximately 0.4 mm (Figure 3).

**Figure 4:**
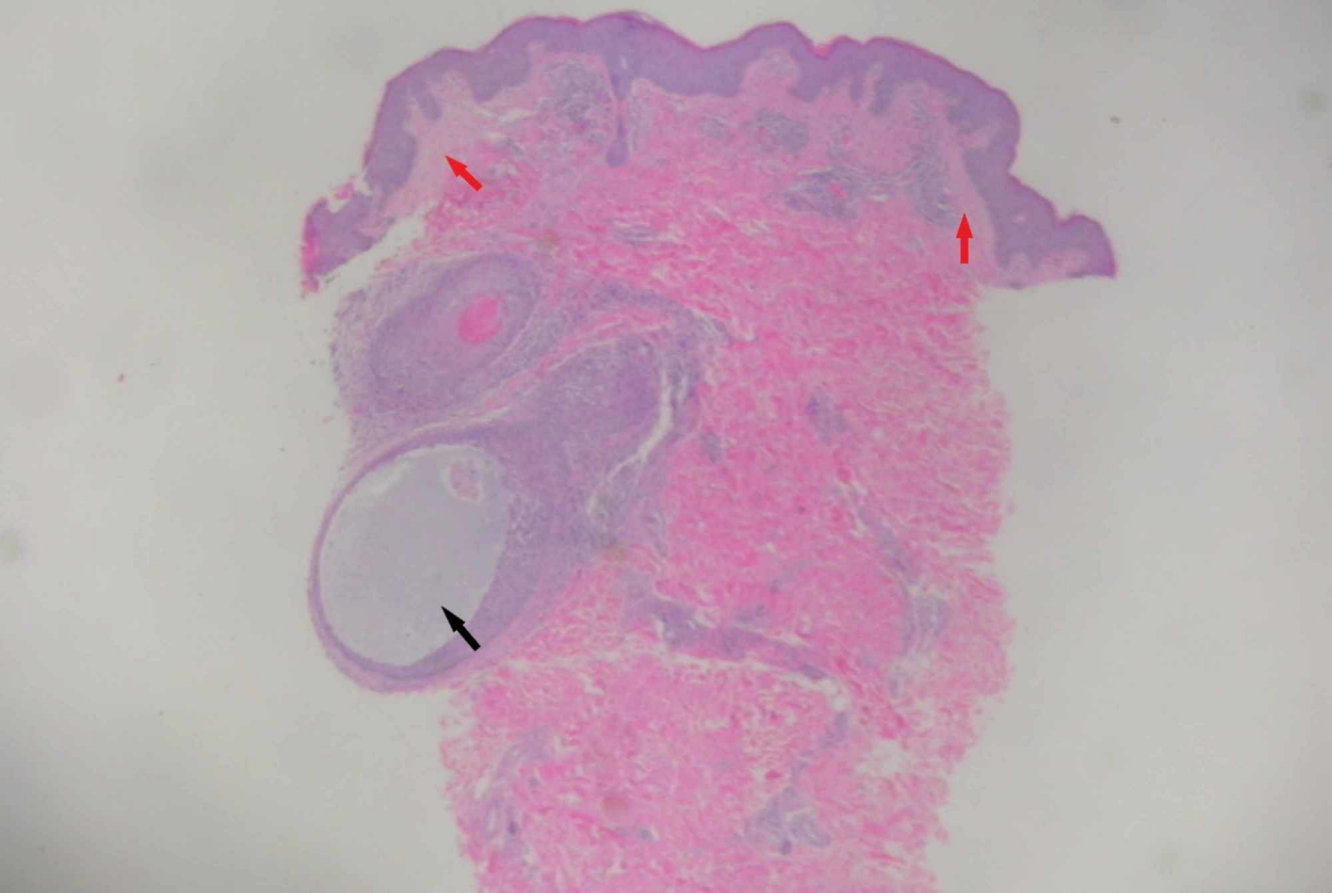
Histological section showing minimal edema in the upper dermis (red arrows) and accumulation of mucin within the hair follicle (black arrow).

**Figure 5:**
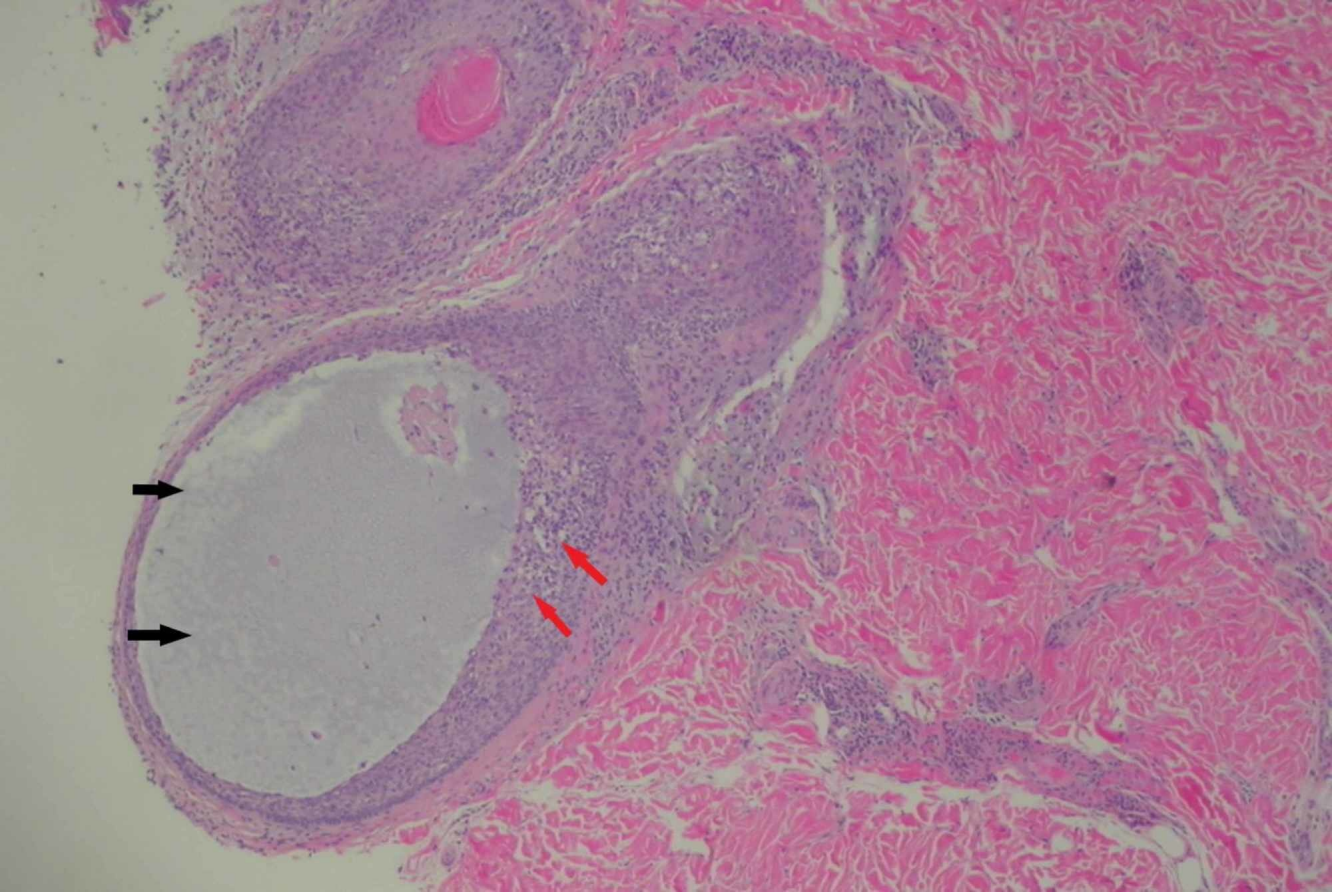
Histological section showing a degenerated follicle filled with mucin (black arrows) with perifollicular chronic infiltrates (red arrows).

A 3-mm punch biopsy was performed and showed mucinous degeneration of the hair follicles and perifollicular chronic inflammation with no atypical cytological features. The upper dermis showed slight edema with minimal lymphocytic infiltrates. The overlying epidermis showed no significant abnormalities with no obvious spongiosis or hyperkeratosis (Figures 4-5). Other routine laboratory investigations were essentially normal.

The diagnosis of FM was established. This young woman was managed with topical steroids, phototherapy (natural sunlight), hydroxychloroquine, and emollients for symptomatic relief. At 4 months followup, there was progressive improvement of symptoms with only mild residual papules of the elbows and buttocks.

## Discussion

Follicular mucinosis (also referred to as alopecia mucinosa) was first described by Pinkus as alopecia caused by follicular degeneration secondary to the accumulation of mucin around the outer hair sheath and sebaceous gland, with prominent follicular infiltration by chronic inflammatory cells [3]. Subsequent evidence revealed that this unique degeneration of the pilosebaceaous unit can occur in the presence or absence of alopecia, hence the name FM [4-5]. The pathogenesis of the disease remains largely unknown. It has been postulated to arise from cellular alterations in the affected structures leading to the production of mucin [6]. A cell-mediated immune mechanism as evidenced by a large number of T-cells and macrophages, with markedly increased number of Langerhans cells within the affected follicle has also been suggested to play a role [7].

It can be broadly classified into three different forms with variations in onset, course, and disease associations. The first is a primary acute form which occurs more commonly in children and younger adults, with solitary lesions seen on the head and scalp that resolve spontaneously within a relatively short period. It could also manifest as a primary chronic form which is seen in a slightly older age group and runs a more protracted course with multiple disseminated lesions that tend to recur frequently following treatment. The third variant occurs secondary to a wide range of benign (lupus erythematosus, hypertrophic lichen planus, alopecia areata) and malignant (Hodgkin’s lymphoma, leukemia cutis, cutaneous T-cell lymphoma) disorders, the most documented being mycosis fungoides [6, 8]. In this case, a temporal association was believed to exist between the onset of symptoms and the use of gonadotropin releasing hormone antagonists. A review of the literature showed another case in which the similar findings were observed, although in a much older age group [9]. As such, a history of use of such medications should be elicited in various patients with this condition, as this could also point at another possible etiology.

Histologically, the presence of inflammatory infiltrates confined to the perifollicular or perivascular zones, an absence of atypical cells and minimal to no extension of infiltrates to the epidermis or upper portions of the hair follicle are indicative of a more benign condition. On the other hand, involvement of the upper dermis and epidermis, cytological atypia, and the presence of band-like infiltrates are more commonly seen in association with mycosis fungoides [10]. Solitary lesions are also more likely to be benign, though have been reported in FM associated with secondary malignancies [11]. There is, however, no clear cut diagnostic criteria to different benign from more malignant disease processes. Studies into various clinical and immunohistochemical methods to achieve this have shown significant overlap in the features of both primary and secondary forms of the disease, with none being able to reliably predict disease progression and subsequent outcomes in the affected individuals [12-13].

Treatment modalities that have been described in the management of this condition include topical, intralesional and systemic glucocorticoids, x-irradiation, dapsone, antimalarials, indomethacin, isotretinoin, minocycline, PUVA photochemotheraphy, and UVA1 phototheraphy [2]. In cases of secondary FM, treatment of the underlying cause leads to resolution of symptoms. The disease has been reported to run a more benign course in children even when associated with mycosis fungoides, with some authors suggesting that a less aggressive approach be used in both diagnosis and treatment of this age group [1, 10]. Expectant management is an option in cases of primary FM as most cases resolve spontaneously within two months to two years. However, the lack of clear cut diagnostic methods and the potential association of FM with other more aggressive disease conditions warrant a need for constant followup usually for a minimum of five years in affected individuals to enable early detection of signs of other malignancies [8, 14].

## Conclusions

Follicular mucinosis is a rare skin disorder with characteristic histological findings, but with a varied clinical course and multiple disease associations. Extensive involvement as seen in our patient is rare and though spontaneous resolution is the norm with most primary benign cases, adequate followup with long-term surveillance remains a mainstay in the management of this condition to ensure early identification of possible malignancies and to reduce adverse outcomes.
